# Mechanical Aspects of Angiogenesis

**DOI:** 10.3390/cancers13194987

**Published:** 2021-10-05

**Authors:** Maibritt Kretschmer, Daniel Rüdiger, Stefan Zahler

**Affiliations:** Department of Pharmacy, Pharmaceutical Biology, Ludwig-Maximilians-Universität München, Butenandtstraße 5-13, 81377 Munich, Germany; maibritt.kretschmer@cup.uni-muenchen.de (M.K.); daniel.ruediger@cup.uni-muenchen.de (D.R.)

**Keywords:** angiogenesis, ECM, mechanical cues, stiffness, endothelial cells, tip/stalk cells, cell migration, vessel stabilization, tumor angiogenesis, cancer treatment

## Abstract

**Simple Summary:**

The formation of new blood vessels from already existing ones is a process of high clinical relevance, since it is of great importance for both physiological and pathological processes. In regard to tumors, the process is crucial, since it ensures the supply with nutrients and the growth of the tumor. The influence of mechanical factors on this biological process is an emerging field. Until now, the shear force of the blood flow has been considered the main mechanical parameter during angiogenesis. This review article provides an overview of further mechanical cues, with particular focus on the surrounding extracellular matrix impacting the cell behavior and, thus, regulating angiogenesis. This underlines the enormous importance of the mechanical properties of the extracellular matrix on cell biological processes and shows how changing the mechanics of the extracellular matrix could be used as a possible therapeutic approach in cancer therapy.

**Abstract:**

Angiogenesis is of high clinical relevance as it plays a crucial role in physiological (e.g., tissue regeneration) and pathological processes (e.g., tumor growth). Besides chemical signals, such as VEGF, the relationship between cells and the extracellular matrix (ECM) can influence endothelial cell behavior during angiogenesis. Previously, in terms of the connection between angiogenesis and mechanical factors, researchers have focused on shear forces due to blood flow. However, it is becoming increasingly important to include the direct influence of the ECM on biological processes, such as angiogenesis. In this context, we focus on the stiffness of the surrounding ECM and the adhesion of cells to the ECM. Furthermore, we highlight the mechanical cues during the main stages of angiogenesis: cell migration, tip and stalk cells, and vessel stabilization. It becomes clear that the different stages of angiogenesis require various chemical and mechanical cues to be modulated by/modulate the stiffness of the ECM. Thus, changes of the ECM during tumor growth represent additional potential dysregulations of angiogenesis in addition to erroneous biochemical signals. This awareness could be the basis of therapeutic approaches to counteract specific processes in tumor angiogenesis.

## 1. Introduction

Sprouting angiogenesis is the formation of new blood vessels from pre-existing ones and plays a role in physiological processes, such as tissue regeneration, wound healing, embryonic development, and morphogenesis, as well as in pathological processes, such as tumor growth and metastasis [1,2,3]. The development of various organs is significantly determined by angiogenesis [4]. The role of angiogenesis in tumor growth is equally important [1,3,5], which makes it one of the hallmarks of cancer [6]. Accordingly, the understanding of these processes is of great scientific and clinical importance. Vascular endothelial growth factor (VEGF) is undoubtedly a central player in the process that forms and drives the vascular network through chemotactic gradients [1,2,3]. In 1971, Folkman was the first to recognize tumor angiogenesis as a putative therapeutic target [7], and over the years, the clinical success of anti-VEGF therapy developed [1,3].

However, in recent years, it has become increasingly clear that biomechanical cues and mechanical interactions of cells with the extracellular matrix (ECM) also influence vascular development and shape organ specific vascular beds [8,9]. Mechanosensing allows cells to convert mechanical signals intracellularly into biochemical ones and adapt their cell behavior. In the context of angiogenesis, biomechanical influences have been studied, mainly from the point of view of shear stress generated by blood flow. Several reviews have already been published on this topic [4,10,11,12].

Tumor formation is associated with biomechanical changes of the microenvironment [9,13]. For tumor biology, stiffness resulting from the extracellular matrix (ECM) is of great importance. On the one hand, tumor development can be supported by an initial increase in stiffness, because of chronic inflammation or fibrosis [8,14]. On the other hand, tumor development by itself causes an increase in stiffness [8,13]. For this reason, tumor areas can be identified by an increased stiffness compared to the surrounding tissue. This results in a highly variable and changing stiffness of the ECM. Therefore, in addition to shear stress, the influence of stiffness on endothelial cells is of great importance. This relates to tumor angiogenesis as well as to physiological angiogenesis, since different organs also show different stiffness ranges.

**Figure 1:**
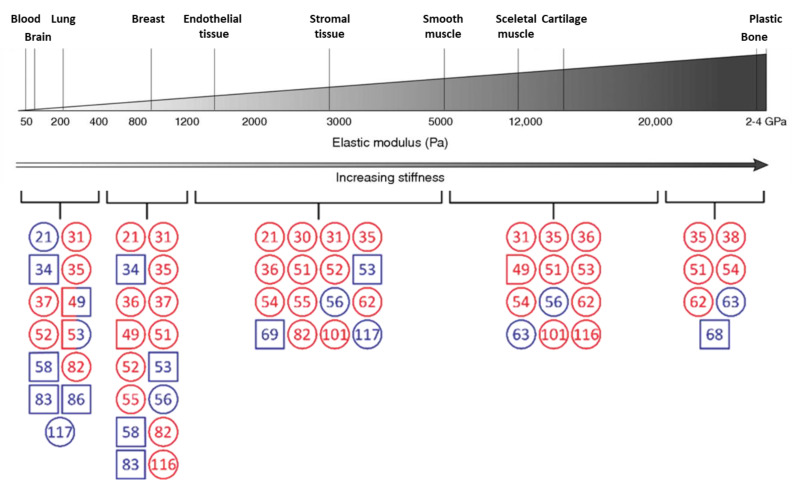
Summary of referenced stiffnesses in relation to different tissue stiffnesses (adapted from [13]). The data from the referenced literature cover a wide range of stiffnesses. Stiffness was divided into five ranges: up to 400 Pa, 400–1200 Pa, 1200–5000 Pa, 5000–20,000 Pa, and greater than 20,000 Pa. For each range, the references that investigated the corresponding stiffness value were listed. The listed stiffnesses of the references relate to in vitro experiments with endothelial cells. For references in blue, natural gels (e.g., collagen, Matrigel, fibrin) were used, while for references in red, the measured stiffness is related to synthetic gels (e.g., polyacrylamide, polydimethylsiloxane). Mixed colors show that both natural and synthetic gels have been investigated in this stiffness range. References in a circle indicate 2D models and references in a square indicate 3D models, while for hybrid shapes, both 2D and 3D experiments were performed for the respective stiffness range. Numbers within icons refer to references.

The aim of this review is to provide an overview of how the stiffness of the ECM affects endothelial cell behavior, based on the essential steps of angiogenesis: cell migration, tip/stalk cell selection, and vessel stabilization. The focus lies on the influence of stiffness; for the overview on the architecture of blood vessels, the ECM, and the basement membrane, we refer to other reviews [15,16]. Figure 1 lists published stiffness values for various organs that have been measured to date (related information on the stiffness of healthy and diseased tissues and organs are summarized in the review by Zanotelli and Reinhart-King [5]), and sets them into context with matrices, which have been used to study endothelial cell behavior. Table 1 provides an overview of the factors and their regulating processes that influence ECM stiffness.

## 2. Cell-Matrix Contacts

In order for cells to react to (or to be influenced by) the stiffness of the ECM, a cell-matrix contact is a fundamental requirement. Inhibition of adhesion at the beginning of the tube formation in vitro completely prevents the formation of a network [17]. Adhesion is not only influenced by the type of matrix protein, density, concentration, and topography, but also by the stiffness of the ECM. The stiffness affects the adhesion surface and can regulate cell processes.

Different ECM proteins or binding sites differentially affect adhesion [18], spreading [18,19], migration [18,19], and proliferation of endothelial cells [20]. Their presence is a prerequisite for a certain cell behavior (e.g., the presence of laminin is necessary for tube formation [17,20,21]). Individual proteins can have positive effects on angiogenesis, such as the enhancement of sprouting by collagen IV [22], or negative effects, such as the disruption of tube formation by collagen I [21,23]. Furthermore, different ECM proteins in combination with the respective integrin binding partner affect intracellular signaling pathways and cell behavior. Collagen I activates certain key regulators for actin reorganization in contrast to laminin-111 [24]. The review by Davis and Senger provides a good overview of different signaling pathways by the binding of different integrins [25]. As the focus of the review is on mechanical influence, we refer to other reviews for the effect of ECM proteins [26] or ECM protein fragments [27] in connection with endothelial cells.

In addition to the type of protein, topography, density, and concentration also influence cell behavior [18,28,29,30]. A low concentration leads to a reduction in ligand density and cell–ECM contact [31], while a higher concentration leads to faster and more focal adhesion [30]. The change in concentration also leads to a change in mechanical resistance [32,33]. Therefore, the stiffness can also influence cell adhesion and cell–ECM contact. In the literature, there are, however, controversial results. The increase in spreading and adhesion area or cell area for a stiffness range from 500 to 10,000 Pa [34,35,36,37], and the decrease in adhesion [38], have been described with increasing stiffness. In contrast, the reduction of adhesion is described for very high stiffness between 25,000 and 75,000 Pa. Further investigations are needed to determine the exact effect of stiffness on adhesion. In this context, it is also unclear exactly how cells probe the stiffness of the ECM (cells might exert constant stress and react to the degree of strain or exert a constant deformation and monitor the required stress [39]).

If the adhesion is too low, this leads to apoptosis. With medium adhesion, the cells differentiate, while firm adhesion leads to cell proliferation [40]. Excessive adhesion disturbs tube formation [23] and sprouting [33]. Therefore, a balanced adhesion is crucial for optimum cell processes [18]. Due to the combination of adhesion surface and stiffness, a balanced stiffness is also necessary. In addition, ECM stiffness at the adhesion surface influences the translocation of the mechanosensitive transcription factors MRTF-A and YAP [41], and the general gene expression for genes associated with angiogenesis [33].

## 3. Cell Migration

Migration of endothelial cells into surrounding tissues is an important step for the formation of new blood vessels during angiogenesis. A rise in stiffness supports migration and ECM remodeling via increasing contraction and traction force. Stiffness gradients support directed migration. However, if the stiffness is too high, a mechanical barrier is created and the stiffness has a negative effect on migration. The review by Lamalice, Le Boeuf, and Huot provides an overview of endothelial cell migration in angiogenesis in general [42]. In the following, we will focus on the contribution of stiffness.

As already described, stiffness influences the adhesion area. With increasing adhesion, migration also increases [43,44], whereby excessive adhesion again has a negative effect on migration [18]. At very low adhesion, endothelial cells exhibit membrane blebbing during migration [44]. The effect of adhesion is also reflected in the different migration behavior depending on the dimensionality of the experiments [44].

Similar to what was described above for adhesion—the type of ECM molecules [19,21,45] and topography [29] also affect extent and mode of migration. The blocking of integrin binding sites for laminin causes a morphology switch from elongated to round with blebs [45]. Increased migration is associated with collagen I [21,45]. In this latter case, cells align along the collagen network [46,47] and follow the stiffer collagen fibers [21]. This was shown in 2D and 3D experiments as well. In addition, migration is increased by formation of lamellipodia, for example by laminin-411 [19], and migration speed is regulated by adhesion-dependent signals [48].

In addition, stiffness has a direct influence on migration. Increased stiffness between 500 and 2500 Pa leads to increased migration of the cells [49,50,51,52,53] and an increase in sprouting [34,53,54]. This is slightly in contrast to the results that tube formation is reduced with increased stiffness [23,32]. Network formation can be completely prevented when the stiffness is too high (over 4000 Pa) [21,31,52,54,55,56]. However, if the stiffness is too low, the tube formation is completely inhibited [31]. In contrast, some works show that sprouting is reduced with increased stiffness [33,57]. It seems that the optimum stiffness for migration has to be balanced with adhesion. This also makes sense from the point of view that an increase in stiffness or concentration of ECM proteins represent an increase in the mechanical barrier [32,53], which is, of course, negative for sprouting [53,58,59,60].

It has been postulated that tube formation is determined by a balance of cell traction and mechanical resistance [32]. Along this line, the largest traction force was measured at the sprout ends [61], and increases with the rising adhesion area [30,36]. In addition to the indirect influence of the stiffness on the traction force via the adhesion surface, the stiffness also has a direct influence. An increase in stiffness leads to an increase in traction force [36,62]. Since traction force is generated by cell contractility [55,58], stiffness also affects actin structure. As stiffness increases, more actin fibers are formed in the cells [62,63] and cell stiffness increases [54]. The generation of actin stress fibers is favored by collagen in contrast to laminin-111 [24]. However, it was shown that the formation of stress fibers is not excessive in both tube formation [23] and migration in collagen I or Matrigel [45]. This illustrates that an excessively increased stiffness is not advantageous, which is also underlined by the fact that, with a rise in contraction, the adhesion area decreases and, thus, has a negative effect on migration [44].

The range of the traction force determines how far cells can communicate [51]. Accordingly, inhibition of contraction reduces sprouting length [57]. Traction force and contraction provide a realignment of the ECM [21,47,56,64,65], resulting in an increase in stiffness [21,58], and the cells follow this self-made stiffness gradient (migration by durotaxis) [21]. In the same manner, it has been shown that cell migration [66,67], sprouting [66,68,69], and vessel alignment [46,68] can be oriented and enhanced by the ECM alignment due to cyclic strain. However, it is not exactly clear whether the cells follow the stiffness gradient or the orientation of the ECM and a density gradient of ECM proteins (migration by haptotaxis). In this context, it was observed that if the density of the ECM or the stiffness is too high (between 1000 and 4000 Pa), remodeling is reduced or completely inhibited [21,56]. The relationship among traction force, ECM track formation, ECM remodeling, and cell behavior was previously summarized in several reviews [11,25,70,71].

Cell migration is supported with increasing stiffness. However, a negative feedback loop exists, since when the stiffness becomes too large, the necessary contraction force becomes too large, causing the adhesion area to decrease again and, consequently, migration is reduced. In addition, the mechanical barrier increases with higher stiffness and ECM remodeling as a migration supporting process is inhibited. The migration process occurs as a constant alternation between strong cell contraction with low adhesion for ECM remodeling and reduced contraction with increased adhesion to support migration along a created gradient. To maintain a balanced stiffness and no excessive increase, the cells use two processes. On the one hand, endothelial cells synthesize ECM proteins [31,45,56] and crosslinking enzymes [50], which affect the structure and mechanics of the ECM. This plays a role in tube formation [72] as well as in migration [73]. On the other hand, with increasing stiffness, the activity of matrix metalloproteases also increases [53]. This reduces contraction [65] and creates a balance between matrix tension and proteolysis [47]. Protease systems, mainly the urokinase-type plasminogen activator (uPA) and the matrix metalloproteases (MMPs), generally play important roles in angiogenesis for degradation of the basement membrane [45,74,75,76] and invasion of surrounding tissues [77]. The serine proteinase uPA and its receptor uPAR, which play major roles in the fibrinolytic system, activate the pro-MMPs to active MMPs [75,76], thus also modulating ECM architecture. Both uPAR and MMPs are directly associated with tip cells, due to their expression in the filopodia of these migrating cells. After the initial start of cell migration, the selection between tip and stalk cells is the next crucial step in angiogenesis.

## 4. Tip/Stalk Cell Selection

The selection of tip and stalk cells is initially regulated by biochemical signals transmitted by the surrounding tissue [49,78]. For cellular movement and migration into the ECM, initiated by stiffness gradients, however, the biochemical cues are converted into mechanical forces to allow vessel sprouting [49,78,79]. The direction of cell migration is again controlled by chemotactic gradients [80]. The biochemically induced tip stalk cell selection is widely accepted to be under the influence of VEGF and is summarized in detail by Blanco and Gerhardt [2]. Here we will focus on the contribution of the ECM.

The ECM can act on endothelial cells (EC) through biochemical, chemotactic, and mechanical cues and, thus, influences sprout branching and tip, stalk cell differentiation, and vessel formation. Biochemical and chemotactic signals are released from the ECM, whereas the mechanical forces are generated by changes in the matrix density and elasticity [81,82,83]. The ECM density regulates the morphology and, thus, the proliferation rates of endothelial cells by influencing the cell-matrix adhesions. Increased ECM densities enhance and regulate the matrix entanglement and the cell–ECM connections, leading to higher numbers of proliferating cells compared to migrating cells [49,81,83]. Proliferation is a characteristic of stalk cells, allowing sprout formation and elongation [82,83]. Proliferation rates are regulated by mechanical stretch. High degrees of stretch induced by cell morphological changes via matrix stiffness increase endothelial proliferation and reduce sprout formation [80]. Consequently, in order for a tip cell to break out of an existing vessel, the ECM elasticity has to be increased so that cell-matrix connections are loosened and the cell can proliferate due to less mechanical stretch. Further changes in matrix density, as well as matrix remodeling, are required to allow the tip cells to protrude into the ECM [33,49]. The matrix deformation and remodeling is regulated by tip cell contractility. Cell contractility is dependent on myosin II activity and translocation as well as ECM stiffness [21,49]. Cellular contractile forces are obtained by translocation of the actin-binding non-muscle protein myosin II to the EC cortex leading to cortical contraction [49]. Contracted endothelial tip cells are able to deposit ECM proteins arranging stiffened matrix tracks that promote tip cell migration [21]. During further EC branching, the protruded ECM is degraded by MMPs leading to local density reduction and subsequent remodeling promoting tip cell migration [33,49]. This ECM degradation is largely mediated by the uPA/uPAR system. The cell surface receptor uPAR that is located on the leading edge of the migrating ECs binds to the serine proteinase uPA and activates it. Activated uPA in turn mediates the plasmin-dependent proteolysis that activates the MMPs cascade [74,76,84]. The uPA/uPAR system itself is initiated via biochemical signaling by pro-angiogenic growth factors, such as VEGF, redistributing the uPA receptor on the cell surface to the migration edge of the cell, and driving directed proteolysis [76,85]. Matrix degeneration and sprout elongation is directed by filopodia branching from the tip cells connecting with the ECM [49,86]. Filopodia extension is further mediated by downregulation of myosin II induced cell contraction [49]. Mechanical impacts, applied by the filopodia, support tip cell intrusion into the matrix via pulling and pushing forces [49,86]. While the cell-matrix adhesion of the migrating tip cells is reduced by MMP-induced ECM degradation, the matrix subsequently has to be stiffened again by deposition of the ECM proteins in order to enable adhesion and proliferation of the following stalk cells. Hence, a stiffness gradient of the matrix and, thus, an adhesion gradient is necessary for a controlled sprouting and sprout extension [33].

Cell-matrix adhesions are additionally influenced by endothelial integrin receptors interacting with ECM proteins and mediating focal adhesion, again regulating EC proliferation and migration [33,81]. The communication between the ECM and the integrin receptors is influenced by VEGF regulating the ECM structure as well as the tip and stalk cell selection [81]. In addition to VEGF, the uPA/uPAR system also plays a role in integrin-mediated cell-matrix adhesion. This is because the uPA/uPAR system has an intrinsic duality, which enables it to influence cellular adhesiveness in addition to its function as a serine protease [74,84]. VEGF mediated redistribution of the uPA receptor may additionally lead to interaction of uPAR with integrin α5β1 at the focal adhesions of the cellular migration edge [85,87]. Binding of uPAR to integrin results in concomitant reorganization and integrin activation. The uPAR/integrin complex shifts cell-matrix adhesions and may regulate intracellular signaling pathways. This enables cell migration, cell invasion, and uPA/uPAR-driven matrix degradation [76,85,87]. Integrin signaling is furthermore directly affected by VEGF via induction of laminin matrix deposition [81].

The tip and stalk cell selection depends on Notch1-Dll4 lateral inhibition and the Notch signaling pathway [81,88]. The Notch signaling pathway interacts with both the integrin adhesion receptors and VEGF. Expression of the Notch ligand Delta-like 4 is promoted directly by VEGF downstream of VEGFR-2 induction, or by laminin-binding integrins following VEGF-dependent laminin deposition [81,82]. Since the Dll4 expression is activated by biochemical signals as a result of matrix changes, the effect of mechanical forces on the ECs is essential for the regulation of the Notch signaling pathway and, thus, also the tip and stalk cell selection. The stalk cell behavior is regulated by Dll4 and is signified by activation of the Notch signaling pathway in the corresponding stalk cells [81,88,89]. Increased cell-matrix adhesion enhances the stalk cell phenotype by interaction of the Notch intracellular domain with β1 integrin receptors [81]. However, excessive tip cell formation is limited through Notch1-Dll4 lateral inhibition as well as cellular compressive stress [81,88].

Migration and filopodia formation of tip cells, as well as proliferation of stalk cells, are both influenced by the transcriptional co-activators YAP and TAZ through actin cytoskeleton remodeling [90,91]. YAP/TAZ regulate stalk cell proliferation by mechanical stimulation instead of biochemical stimulation via VEGF. Mechanical cues, such as matrix stiffness and cellular stretch, are sensed by YAP/TAZ and translated into biochemical signals, controlling endothelial sprouting and sprout structure [90]. YAP/TAZ further supports stalk cell elongation by extending VE-cadherin turnover at the cell–cell contacts. Increased VE-cadherin-dependent tightening of the cell junctions enables vessel growth and preservation of the endothelial barrier [90,92,93].

## 5. Vessel Stabilization

The final phase of angiogenesis is the maturation and stabilization of the newly formed vessels [94,95]. Vascular maturation includes vessel condensation and alignment via increased cell–cell adhesion, shear stress induced vessel shaping, and external stabilization by cell recruitment [78,94,95,96]. Mechanical cues also maintain pre-existing vessel integrity [97,98].

The blood flow exerts mechanical stimuli on the cells lining the inside of the newly formed vessels. Mechanical stimuli act either as shear stress tangential to the vessel or as mechanical strain transversal to the direction of blood flow on the ECs [98]. Continuous shear stress initiated by the physiological blood flow decreases the VEGF expression, leading to an inhibition of filopodia formation [98,99]. By subsequent inhibition of the EC migration activity and tip cell-induced matrix remodeling the vessel elongation is stopped, and the vessel is stabilized [99]. Further, shear force activates signaling pathways in the ECs resulting in vessel remodeling and cell arrangement. Vessel extension is being advanced so that the thick vessels branch out into thinner ones [78]. Oscillatory mechanical strain, however, deactivates the Hippo pathway, and, consequently, YAP is translocated into the nucleus. Additionally, the cell–cell contacts are tightened by increased β-catenin expression levels, altogether leading to elevated ECM expression around the newly formed vessel [97]. The oscillatory stretch also stimulates mural cell recruitment and differentiation, hence the mural cells express stabilizing growth factors supporting vessel maturation [97].

Endothelial cell–cell connections are crucial for the stabilization of new blood vessels in the angiogenesis process. Like cell-matrix contacts, cell–cell contacts are also influenced by certain ECM proteins, and, consequently, stiffness dependent. For example, the presence of laminin β1 chains increases cell–cell contacts in tube formation [17]. In contrast, collagen I ensures the disruption of cell–cell connections [24]. This is of note, since collagen I forms the main component of many surrounding tissues and, thus, promotes the migration behavior of the cells. Fibronectin, which is essential for cell–cell contacts of endothelial cells, is also of particular importance [31,100]. Without fibronectin, cells would grow and move on top of each other [100]. Fibronectin is mainly synthesized by the cells themselves and influences the cytoskeleton as well as enhances the levels of the adhesion protein VE-cadherin [100]. The formation of VE-cadherin cell–cell contacts is also influenced by the uPA/uPAR system. Upregulation of uPA/uPAR, mediated by VEGF at the onset of angiogenesis leads to increased degradation of VE-cadherin contacts and, thus, prevents the formation of new ones [85]. To block the catalytic activity of uPA/uPAR and to enable vessel stabilization, the plasminogen activator inhibitor PAI-1 is released by the ECs. PAI-1 prevents the formation of plasmin, thereby interrupting the uPA/uPAR system and inhibiting growth factor expression by the ECM as well as ECM and cell–cell contact degradation [75]. VE-cadherin also has a major influence on sealing the newly formed vessel [56,97]. Without VE-cadherin, a stable network cannot develop and the EC tube disintegrates into single cells [56]. In addition to fibronectin, stiffness influences cell–cell connections and VE-cadherins. However, experimental evidence is currently rather limited and further research is necessary in this field. In tendency, increasing stiffness for the range of 500 to 33,000 Pa seems to reduce cell–cell contacts [51,56,101]. This leads to increased dispersion of cells and increased permeability of the endothelial cell layer [51,101]. A possible explanation is the increasing contraction of the cells with rising stiffness [62,101]. As a result, the cell connections may be weakened [62,101]. Nevertheless, other studies, which investigate the same range (200–20,000 Pa), show that a stiffness increase enhances cell–cell contacts and the cells adhere together to from a connected network [53,63]. Bordeleau et al. also describes enhanced cell–cell contacts with increased permeability [53]. This again underlines the contradictory and inconclusive states of research concerning the effect of stiffness on endothelial cell contacts. However, not only do the endothelial cell connections play a role in stabilizing newly formed vessels, but also the interactions between the endothelial cells and recruited mural cells is essential for the support and maintenance of the newly formed vessel [102,103].

The recruitment of mural cells like pericytes and vascular smooth muscle cells support vessel stabilization by mechanical stimulation [96,97,104]. The perivascular cells promote vessel maturation in addition through paracrine signaling [105]. While the accumulation of mural cells is increased, the proliferation in the ECs is decreased [97,102,104]. The cell recruitment further regulates the ECM development and composition, increasing vessel stability, whereas the matrix alignment is also supported by mechanical stretch stimulus [97,106]. Pericyte–EC interaction leads to prevention of proteolysis as well as an increased integrin expression of the pericytes, allowing for condensation of the newly formed ECM [106]. Disintegration of the matrix structure is prevented by the inhibition of certain matrix metalloproteases, which would otherwise allow ECs to penetrate the ECM [106]. Pericytes, in particular, also contribute to vessel stabilization via secretion of signaling mediators, and contact-dependent signaling [102,103,105]. However, pericytes also affect the vessel by exerting contractile forces. The mechanical cues can deform and condense the matrix, thus influencing EC cohesion of the vessel [102]. The interaction between ECs and pericytes not only influences vessel maturation, but also regulates the degeneration of existing vessels through separation of the EC-pericyte connection [103]. Accordingly, a new vessel sprouting starts with the detachment of the pericytes from the EC tube and, thus, reactivates the migration of the tip cells and the proliferation of the stalk cells [96]. In addition to the pericytes, fibroblasts, mesenchymal stem cells, myeloid cells, and various inflammatory cells also stabilize the newly formed blood vessels [97,105,107]. The fibroblasts and mesenchymal stem cells in particular support the vessel through direct attachment, while circulating myeloid cells and inflammatory cells are recruited to the relevant sites, and contribute to stabilization and maturation through mechanical support, as well as biochemical signaling [105,107].

The ECM surrounding the EC tube and its supporting cells control vessel integrity by increasing the responsiveness to mechanical strain exerted by the blood flow via signal secretion [97,104]. The mere recruitment of pericytes serves to support the vessel, but does not yet enable coherence and resistance to mechanical stimuli. In addition to the EC-pericyte interactions, the expression of cell adhesion molecules, such as cadherins initiated by the surrounding matrix, also contribute to vessel maturation and stabilization [108,109]. The major adhesion molecule fibronectin, derived by the ECs of the tube, initiates integrin expression in the ECs [104]. Interaction between the ECM and integrin then enables stabilization and cohesion of the neo-vessel as well as the connection of ECs and mural cells [104,110]. While the regulation of these adhesion molecules is controlled by ECM accumulation, remodeling and, consequently, stiffness, a lack of expression of the matrix molecules would prevent cell recruitment and continued tube formation [108]. Inhibition of the integrin mediated EC-mural cell adhesion would further cause vessel decay as well as apoptosis in both ECs and mural cells [110]. The cell adhesion molecules also enable the maintenance of the vessels and the resilience against mechanical stress [94]. Degeneration of the ECM, which is important for the new vessel formation, is prevented by the blockage of collagen lysis at this stage [111]. The linked compression of the collagen fibers in the matrix also serves to stabilize the vessel, as the collagen deposition increases the stiffness in the ECM. The EC tube support further occurs by tight binding of the ECs to the collagen fibrils of the matrix [111]. Disintegration of the matrix and the related destabilization of the neo-vessels is inhibited by MMP activity control [94,106,111]. Increased proteolysis of matrix components by MMPs, which proceeds downstream of EC-pericyte interactions, can lead to the decay of the ECM and, consequently, also to the damage of the ECs [94,111]. MMP dependent severing of cell–cell as well as cell-matrix connections impairs vessel stabilization and maturation equally through disturbance of mechanical adhesion [94]. Regulation of MMP activity depends on the tissue inhibitor of metalloprotease TIMP [106,111]. The MMP inhibitor especially interferes with lysis of collagen and compression of the collagen fibers, maintaining the integrity of the ECM and the neo-vessel [94,111].

Moreover, the YAP/TAZ pathway also influences the resistance of newly formed vessels to mechanical strain [97]. In particular, YAP1 regulates vascular lumen support upon its translocation into the nuclei of the ECs in response to the shear forces of the bloodstream [97,98]. This nuclear translocation can be inhibited again by VE-cadherin mediated cell–cell contacts [41]. In the recruited fibroblasts that surround the vessel for support, shear stress again induces a nuclear YAP translocation, which increases the proliferation and differentiation of these cells and, thus, improves the mechanical stabilization of the new vessel [97,98].

## 6. Conclusions and Perspective

**Figure 2:**
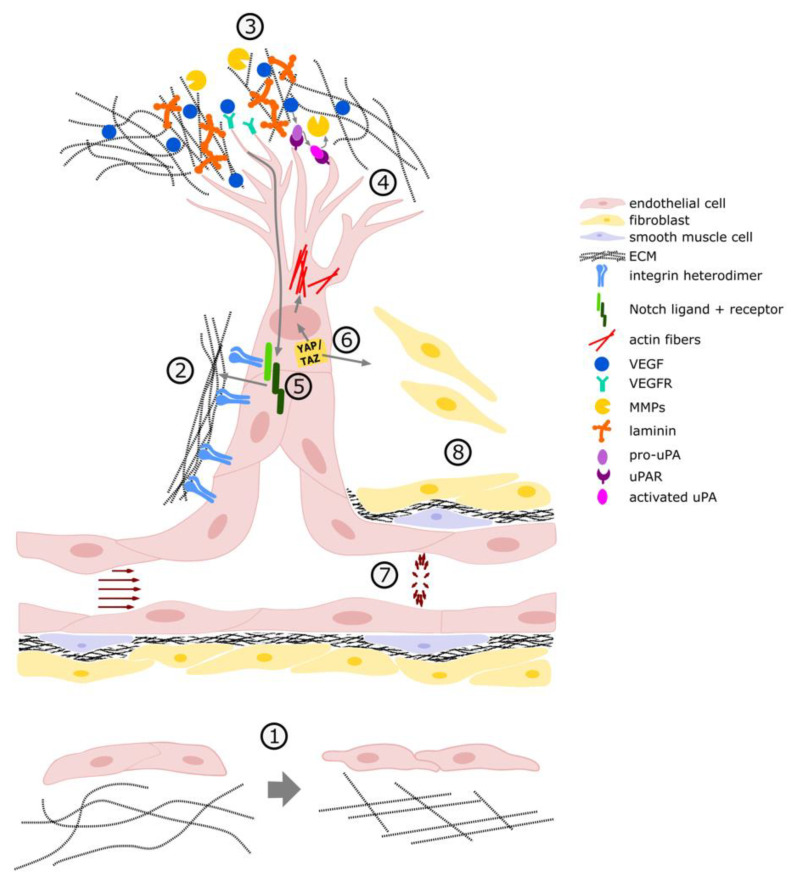
Mechanical aspects of angiogenesis. 1: increased matrix stiffness leads to increased migration; 2: enhanced matrix elasticity (increased stiffness) for tip cell to break out of existing vessel; 3: uPA/uPAR mediated ECM degradation by MMPs; 4: filopodia branching for tip cell direction; 5: VEGF mediated activation of the Notch signaling pathway, increased cell-matrix connections of stalk cells via NICD integrin interactions; 6: activation of YAP/TAZ by mechanical cues influencing the migration and filopodia formation via matrix remodeling, YAP/TAZ further supports ECM formation for vessel stabilization; 7: mechanical stimuli exerted by the blood flow; 8: vessel stabilization by mural cell recruitment.

**Figure 3:**
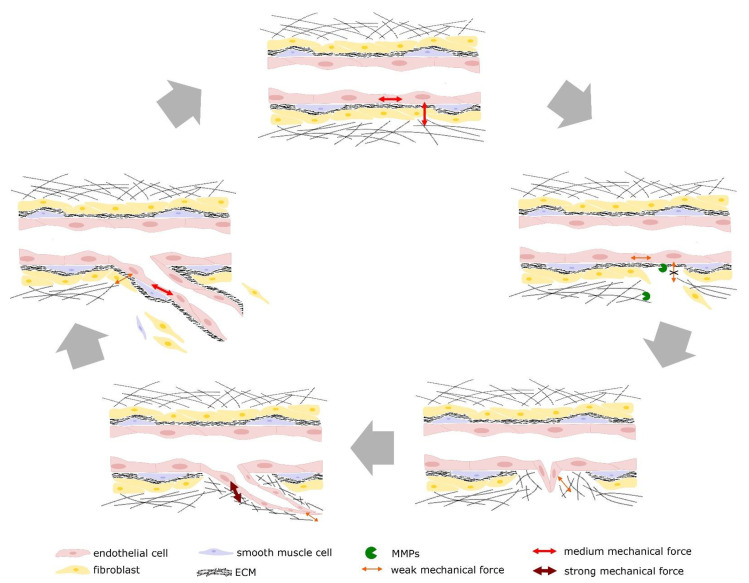
Mechanical forces during angiogenesis. Weak, medium, and strong mechanical forces acting on the ECs from the ECs or between the ECs during the different steps of angiogenesis.

Angiogenesis is largely dependent on biochemical and mechanical signals. Biochemical cues can be translated into mechanical ones or vice versa. The mechanical signals can affect the microenvironment of existing and newly forming blood vessels and activate several signaling pathways [33,78]. Most mechanical changes during angiogenesis occur in the ECM. There, the major biochemical cytokine that initiates the mechanical development is VEGF. VEGF expression leads directly to laminin matrix deposition, inducing local stiffening of the extracellular matrix and creating a stiffness gradient [81,82]. VEGF further induces matrix degradation via uPA/uPAR mediated proteolysis leading to collagen disposition in the ECM and resulting in a stiffness increase [76,85,111]. Additional cytokines and signaling pathways (such as the Notch or YAP/TAZ pathway) may influence matrix properties and other mechanistic aspects of angiogenesis, however, mainly preceded by activation by VEGF or prior matrix alteration. Figure 2 summarizes the different steps of angiogenesis and their relationship between ECM mechanics and cellular behavior. Further, Table 2 and Figure 3 summarize how a change in stiffness affects the various steps of angiogenesis and how strong the forces from the ECM act on the ECs and between the ECs. The starting point and initial key factors of angiogenesis are the contraction, migration, and proliferation of endothelial cells. All of these processes are influenced or regulated by the composition and the density of the ECM via cell–ECM interactions [49,82,83]. By changing the properties of the matrix, the cell-matrix connections are altered, which then affects the behavior of the cells. However, the cells also have a direct effect on the ECM, for example through tip cell contraction during the formation of a new vascular branch. Furthermore, ECM elasticity is influenced by ECM-specific proteins, such as MMPs, which degrade the matrix to allow vessel growth. MMP-induced matrix degradation is accompanied by matrix stiffening at the penetrated sites [33,49,83]. This results in stiffness gradients and localized stiffening of the ECM, which is essential for the formation of new blood vessels. However, it is not possible to make a precise statement on defined stiffness parameters. In vitro endothelial sprouting assays within collagen matrices performed by Mammoto et al. [82] show that the stiffnesses of the matrix for angiogenesis must be fine-tuned and at an appropriate intermediate level between 800 and 850 Pa, because high matrix densities prevent cell migration from tip cells and low matrix densities weaken the cell matrix connections too much. A dysregulated and, especially, a too low matrix density further ensures that newly formed vessels are not stabilized, and cannot withstand the mechanical stress of the blood flow [82]. Thus, although there are optimized stiffness conditions of the ECM that drive angiogenesis, these depend on the phase of angiogenesis and, more importantly, on the tissue type, because each endothelial cell responds differently to changes in matrix composition, depending on the origin of the cells and the corresponding tissue [5,112]. In all tissues, however, excessive stiffening of the matrix and associated vessel regression and leakage affect a variety of diseases, including fibrosis, cardiovascular disease, rheumatoid arthritis, as well as cancer and metastasis [5,112,113]. In contrast to physiological angiogenesis, pathogenic angiogenesis results in irregularly branched and leaky vessels that do not ensure vascular perfusion of the tissue. The abnormal vessels are caused by dysregulated secretion of cytokines, especially VEGF-A, and subsequent incomplete signaling cascades during angiogenesis [114,115]. In many diseases in which angiogenesis is impaired, and especially in most cancers, VEGF-A is strongly overexpressed. While VEGF-A secretion facilitates mechanical matrix remodeling in the early stages of angiogenesis, leading to cell migration and tip/stalk cell differentiation, downregulation of VEGF-A secretion is suppressed, which would promote further matrix remodeling and drive vessel elongation and stabilization [115]. This results in ineffective sprouting with short, unstabilized vessels that cannot withstand the mechanical forces of blood flow. The ECM during pathogenic angiogenesis has a significantly higher stiffness than physiological ECM due to matrix protein deposition and enhanced cross linking, resulting in reduced compliance of the pathogenic matrix and greater forces acting from the matrix on the cells [5,113]. The dysregulated or absent vessel maturation prevents reverse remodeling of the ECM proteins and stiffness as before initiation of angiogenesis. Much is known about tumor tissue stiffening and its effects on cells, angiogenesis, and tumor progression (especially in cancer). The tumor microenvironment and tumor microvasculature are very different from those in healthy tissue. Tumor tissue exhibits an elevated stiffness due to enhanced collagen deposition and collagen cross-linking proteins secreted by tumor cells, creating an increasing solid stress in the tumor tissue. The density of the tumor ECM does not change, only the stiffness [53,116]. Matrix stiffening affects cell–cell contacts, migration, and proliferation of capillary-forming endothelial cells. Endothelial cells lose the ability to reorganize their actin cytoskeleton in response to stiffness changes, resulting in impaired cell contractility and cell–ECM communication, and leading to the formation of a new phenotype with impaired mechanosensitivity, changed protein expressions, and irregular morphology [35,53,116,117]. The abnormal mechanosensitivity and associated baseline tension in the actin cytoskeleton of the cells in turn affects the ECM by creating a prestressed state in the matrix that can lead to alterations in ECM conformity [117]. Despite the changes in tumor ECM and the effects on cells, angiogenesis or tumorigenesis are possible. However, a malformed vascular structure is evident by deviant branching patterns and tortuous and permeable vessels [53,117]. The stiff matrix increases MMP activity, allowing the vessel to grow, but in addition, VE-cadherin cell–cell junctions are destroyed, which affects vessel integrity and leads to leakage [53]. Due to the permeable and malformed vasculature and the exerted pressure by the tumor solid stress, the interstitial pressure and blood flow also increases, building fluid stress in the tumor tissue [117].

The tensions and forces built-up by the tumor tissue can also condense and affect the surrounding healthy tissue, promoting cancer cell invasion [118]. Further studies also show that the changes in the tumor matrix and the accumulated mechanical forces lead to increased tumor aggressiveness and progression, metastatic potential as well as treatment resistance [116,118].

With the growing understanding of the importance of mechanical influences on angiogenesis, mechanical changes in tumorigenesis, and their influence on tumor progression, new treatment strategies can be developed. The therapy approaches, which have a high clinical potential, have been those preventing or reversing matrix stiffening, or the resulting cellular feedback. Potential targets are the biochemical angiogenesis cues TGFβ and Rho. While TGFβ regulates ECM stiffness through splicing events, the small GTPase Rho and its downstream effector ROCK provide increased cell contractility. Inhibiting both of these targets can lead to normalization of the tumor vasculature and reprograming of the cellular mechanosensitivity, resulting in stabilized and sealed vessels with moderated blood flow [116,117]. In their review, Lampi and Reinhart-King provide an overview of possible FDA-approved drugs that could also be used in the field of reversing pathologic matrix stiffening [113]. The relevance of research into the actuators of pathological matrix stiffening and specifically targeting drugs is increasing. Current anti-angiogenic drugs, which mainly target VEGF, VEGRF2, or inhibit the tyrosine kinase [119], are often used in cancer therapy, but tend to be only transiently active and can quickly lead to a resumption of tumor progression. In this context, the development of new and adapted cell models to study angiogenesis, to identify new targets and to test new drugs plays a major role. Although the importance of ECM stiffness and the associated mechanical forces as well as selective stiffening and softening during angiogenesis are well known, each tissue has a different stiffness and the endothelial cells involved respond differently to the specific mechanical properties. Further, the degree of stiffness change during tumor formation varies in the different tissues (reviewed by Zanotelli and Reinhart-King [5]). Thus, detailed cell models and testing methods should be developed for the better understanding and treatment of cancer, as well as other diseases related to excessive stiffening of the matrix. An optimized model should include not only the tissue-specific endothelial cell type but also the EC vessel-stabilizing cells, such as pericytes and vascular smooth muscle cells, an ECM with corresponding stiffness, the associated ECM proteins and other biochemical cues, as well as a circulating blood stream [112]. Such models could represent the full extent of mechanical influences on angiogenesis, in vitro, and detect all mechanical changes and consequences, giving insight into tissue-specific normal and pathological angiogenesis.

## Figures and Tables

**Table 1:** Factors influencing ECM stiffness and their regulating processes.

Factor	Regulation Process
Kind of ECM protein	Different types of proteins have different basic stiffnesses. Different composition and mixing ratios of different types affect the stiffness. ECM proteins elicit specific signaling events.
Concentration/density/elasticity (local)	Concentration rise increases stiffness and mechanical barrier. Local changes lead to stiffness gradient.
Crosslinking	Increased crosslinking increases stiffness.
Intrinsic tension	A tensioned network has an increased fiber stiffness.
Degradability	Increased degradability provides reduced resistance to proteolysis resulting in faster reduction of stiffness.
Synthesis	Incorporation of additional ECM proteins increases stiffness.
Remodeling	Change of architecture increases tension and stiffness of the ECM network.

**Table 2:** Effect of stiffness modulation on the different steps of angiogenesis. (+ = increased angiogenesis, − = decreased angiogenesis).

Step of Angiogenesis	Stiffness Modulation	Effect on Angiogenesis	Involved Stiffness Factors
Cell-Matrix contact/adhesion	Increased stiffness increases adhesion	+	Kind of ECM protein Concentration
Cell migration	Increased stiffness enhances migration	+	Kind of ECM protein Concentration Intrinsic tension Degradability Remodeling
	Stiffness gradients determine migration direction	+	
	Excessively high stiffness increases the mechanical barrier and reduces migration	−	
Tip/stalk cell selection	Increased stiffness enhances proliferation of stalk cells	+/− (longer sprouts, less branching)	Concentration Synthesis Degradability Remodeling
	Stiffness gradient promotes tip cell migration	+	
Vessel stabilization	Increased stiffness reduces cell–cell contacts and promotes EC permeability	−	Kind of ECM protein Concentration Crosslinking Synthesis Remodeling
	Higher stiffness enhances the mural cell recruitment to support EC	+	
